# A Supervised Contrastive Variational Autoencoder with Probabilistic Latent Alignment for Cross-Domain EEG Emotion Recognition

**DOI:** 10.3390/s26103217

**Published:** 2026-05-19

**Authors:** Linna Wu, Yong Yang, Wenhao Wang, Yuanlun Xie, Nan Zhou, Kaibo Shi

**Affiliations:** 1 School of Electronic Information and Electrical Engineering, Chengdu University, Chengdu 610106, China; 202310306202@cdu.edu.cn (L.W.); xieyuanlun@cdu.edu.cn (Y.X.); zhounan123@cdu.edu.cn (N.Z.); shikaibo@cdu.edu.cn (K.S.); 2 College of Computer Science, Chengdu University, Chengdu 610106, China; 212023081202002@cdu.edu.cn

**Keywords:** EEG-based emotion recognition, domain adaption, variational autoencoder, multi-view supervised contrastive learning

## Abstract

Cross-domain emotion recognition based on electroencephalogram (EEG) is a challenging task, as EEG signals collected from different subjects or at different moments exhibit significant differences in distribution. How to enable deep learning model to learn the common feature space and reduce the distribution differences between the source and target domains is an important research direction. For this problem, we propose a Supervised Contrastive Variational AutoEncoder Network (SCVAE-Net), which possesses enhanced abilities for extracting consistent features across source and target domains, thereby improving cross-domain EEG emotion recognition performance. Specifically, this method utilizes the reconstruction mechanism and latent space probabilization of VAE to obtain intermediate features that are more consistent and transferable. Furthermore, the maximum mean discrepancy loss is employed to further reduce the distribution discrepancy of these features. To alleviate the degradation of discriminative ability during domain alignment, we introduce multi-view supervised contrastive learning in multi-source domains to enhance the intra-class consistency and inter-class separability of latent features. Under the cross-subject and cross-session settings, SCVAE-Net achieves accuracies of 95.01%/96.84% on SEED and 74.94%/79.44% on SEED-IV, respectively. These experimental results demonstrate the effectiveness of the proposed method in cross-domain EEG emotion recognition.

## 1. Introduction

Emotion recognition is a key research direction in human–computer interaction, mental health assessment, and intelligent perception systems [1]. As a signal that can directly reflect brain neural activity, electroencephalogram (EEG) has received widespread attention in emotion recognition research due to its high temporal resolution, non-invasive nature, and sensitivity to changes in emotional states [2,3].

In recent years, with the development of deep learning techniques, EEG-based emotion recognition methods have achieved much progress in automatic feature extraction and end-to-end modelling [4]. Relevant studies employ models such as convolutional neural networks, recurrent neural networks, and Transformers, achieving favourable performance in subject-dependent experiments [5,6,7,8]. For example, Yang et al. [9] propose SGCTNet by integrating the features and topology structure of EEG channels for better recognition performance. In the experiment of Session 1 on the SEED dataset, SGCTNet achieves an accuracy of 94.94 ± 07.44% in the subject-dependent condition. However, it achieves only 70.02 ± 10.09% in the subject-independent condition. This indicates that when these methods are applied to cross-domain scenarios, such as cross-subject and cross-session, their recognition performance often drops significantly. Here, cross-subject refers to training and testing on different individuals, while cross-session refers to training and testing on the same individuals at different times. Both scenarios are more challenging than subject-dependent experiments due to individual differences and temporal variations in EEG signals. Therefore, how to effectively improve the generalization ability of EEG emotion recognition models under cross-domain settings remains an important research question.

The core challenge for cross-domain EEG emotion recognition lies in the highly inconsistent distribution of collected data [2]. On the one hand, there are natural differences in brain structure, physiological status, and emotional response patterns among individuals. Consequently, under identical emotional stimulation, their EEG signals may show much diversity. On the other hand, EEG signals collected from the same subject at different times are frequently influenced by factors such as electrode impedance fluctuations, fatigue levels, and attention fluctuations. This leads to temporal drift in the data distribution. Under the combined effects of these factors, emotion recognition models trained on a single domain often struggle to maintain good performance when applied to unknown subjects or new experiment scenarios.

**Figure 1:**
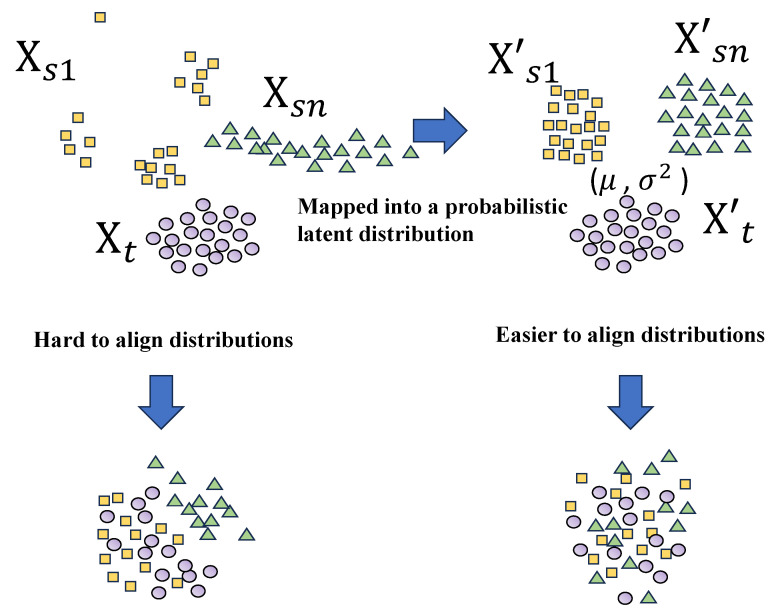
Direct alignment in the original feature space is challenging due to complex distributions, while VAE-based probabilistic modeling yields structured latent distributions that are easier to align.

Therefore, domain adaptation (DA) methods have gradually been introduced into EEG emotion recognition research to alleviate the performance degradation of models under cross-domain problems [10,11]. Existing research mainly reduces the distribution difference between the source and target domains through feature alignment, parameter transfer, and other methods [12,13]. However, these methods still have limitations: Firstly, as shown in Figure 1, most methods rely on directly aligning feature distributions, and when the domains themselves have great noise or individual bias, the adaption is a major challenge. Secondly, some domain alignment methods overly focus on distribution consistency but ignore the discriminative structure between emotional categories, which can easily lead to “inter-class aliasing” of features during the alignment process, thereby weakening classification performance.

Due to its advantages in probabilistic latent representation learning and uncertainty modeling, the variational autoencoder (VAE) has been considered a naturally suitable structure for domain adaptation task structures [14,15]. Introducing it to EEG emotion recognition, its reconstruction learning mechanism can enable it to learn the common feature latent space between EEG signals of the multi-subjects and new subject. Meanwhile, as shown in Figure 1, the probabilistic modeling of potential features and constraining them to follow a Gaussian distribution make it easier for VAE to capture the distribution differences between the source and target domains. Therefore, applying VAE to cross-domain EEG emotion recognition may be a natural and reasonable idea.

Based on this, we propose a Supervised Contrastive Variational AutoEncoder Network (SCVAE-Net) for cross-subject and cross-session EEG emotion recognition. Specifically, this method is designed around how VAE can extract expected latent features. Firstly, we utilize VAE’s reconstruction to learn the potential common space between multi-source domains and target domain. In particular, this is due to numerous studies indicating that different electrode channels have varying degrees of importance for EEG emotion recognition [16,17]. Therefore, we introduce channel attention block in the VAE encoder to enable it to focus on important channels, thus enhancing its feature extraction capability. Then, we introduce the maximum mean discrepancy (MMD) loss into the probabilistic latent spatial features. This further promotes the reduction in feature distribution differences between the source domain and the target domain. However, relying solely on the VAE and MMD constraints merely diminishes the distributional disparity but cannot guarantee the class discriminability of the extracted features. To address this, we further employ a multi-view supervised contrastive learning mechanism to explicitly enhance both the intra-class compactness and inter-class separability of features. It preserves the discriminative structure while aligning the distributions.

The main contributions of this paper can be summarized as follows:

1. We propose a multi-source domain EEG emotion recognition framework, termed SCVAE-Net. By fully exploiting the reconstruction learning and probabilistic latent representation capabilities, the proposed framework is able to effectively capture consistent and transferable features, thereby achieving good performance in cross-domain EEG emotion recognition tasks.
2. Building upon the variational autoencoder’s capability of learning domain-consistent representations, we further introduce the MMD loss to reduce the distribution discrepancy between source and target domains. In addition, a supervised multi-view contrastive learning loss is incorporated to enhance intra-class compactness and inter-class separability of latent features, thereby mitigating the problem of discriminative ability degradation during domain alignment.
3. The proposed SCVAE-Net is extensively evaluated under cross-subject and cross-session settings, achieving 95.01%/96.84% on SEED [18] and 74.94%/79.44% on SEED-IV [19].

In the rest of this paper, Section 2 overviews related work; Section 3 details the proposed method; Section 4 reports the experimental results; Section 5 summarizes the research and future work.

## 2. Related Works

### 2.1. Domain-Adaptive EEG-Based Emotion Recognition

EEG-based emotion recognition is an important direction in affective computing and has attracted widespread attention. Early studies employed traditional methods such as support vector machines and decision trees for recognition. With the development of deep learning, various neural networks such as convolutional neural networks (CNNs), recurrent neural networks (RNNs), and Transformer-based models [5,6,20,21] have emerged. These can automatically learn discriminative representations from EEG signals and demonstrate reasonable performance in subject-dependent experiments. However, due to the low signal-to-noise ratio of EEG signals and substantial inter-individual variability, these methods still have limited generalisability across subjects or sessions. Therefore, existing methods have begun incorporating domain adaptation mechanisms to enhance the model’s cross-domain generalisation capability, mainly including methods based on Generative Adversarial Networks (GANs) and statistical alignment.

Through the adversarial game mechanism of the discriminator, the adversarial network can effectively learn the latent consistent distribution characteristics of complex high-dimensional data across source and target domains, thereby enhancing the model’s ability to characterize data from new domains. Li et al. [10] proposed a Bi-Hemispheric Domain Adversarial Neural Network (BiDANN), which reduces the distributional discrepancy between source and target domains at both global and local levels by introducing a global domain discriminator alongside left and right hemisphere local domain discriminators. Yin et al. [22] proposed a generative cross-subject transfer framework(GITGAN), which achieves feature alignment across subjects through generative transfer and source data selection. This approach enhances EEG cross-subject recognition performance without disrupting the target domain distribution. Li et al. [11] proposed an Emotion Domain Adversarial Neural Network (EDANN), which achieves cross-subject and cross-session alignment of EEG emotional features by introducing an adversarial learning mechanism comprising an encoder, emotion classifier, and domain discriminator. This approach extracts discriminative and domain-invariant emotional representations. Ju et al. [23] proposed a Domain Adversarial Network (DANN-MAT) based on multiple adversarial tasks, which removes emotion-irrelevant subject-dependent variations through multiple adversarial constraints. Sun et al. [24] introduced a domain adversarial transfer network incorporating attention mechanisms. By extracting multi-level features to obtain multi-scale EEG representations, it combines MK-MMD with adversarial learning to achieve global and subdomain alignment, thereby mitigating distribution shifts caused by individual variations.

Statistical alignment methods are widely used in domain adaptation due to their advantages in characterizing data distribution differences and reducing inter-domain biases. For the issue of significant inter-subject variability in EEG data, Hang et al. [12] proposed a deep domain adaptation network for cross-subject EEG recognition. This approach employs convolutional neural networks for feature extraction, utilizes MMD for aligning source and target domain distributions, and incorporates a category-centred discriminative constraint to enhance feature separability. Sun et al. [24] proposed an adversarial domain adaptation emotion recognition model incorporating an attention mechanism. By extracting multi-level features to obtain multi-scale EEG representations, it employs MK-MMD and adversarial learning to achieve cross-subject feature alignment, thereby enhancing the generalisation performance of emotion recognition. Zhong et al. [13] also proposed a deep domain adaptation framework based on correlation alignment (DDAF-CORAL), which utilizes a two-stage deep feature extraction process and aligns the covariances of the source and target domains in the feature space, optimizing both classification performance and domain-invariant representation learning capabilities. Tang et al. [25] proposed an unsupervised domain adaptation framework based on dynamic distribution alignment (UDA-DDA). This method combines MMD and conditional MMD to simultaneously align the marginal and conditional distributions of the source and target domains, and it introduces a pseudo-label confidence filtering mechanism to dynamically optimize the alignment process.

### 2.2. Variational Autoencoder

Variational autoencoder (VAE) is widely used in image generation, denoising, and representation learning [26], as well as speech and time series analysis, due to its powerful probabilistic modeling capabilities and latent representation learning abilities. For example, to address the challenges of data sparsity and insufficient user preference modelling in cross-domain recommendation, Zhang et al. [15] proposed a Cross-Domain Recommendation Variational Autoencoder (CDRVAE) framework. This approach employs VAE as its backbone architecture to model both intra-domain user preferences and cross-domain knowledge transfer. Through an asymmetric decoding structure and cross-domain reconstruction mechanism, it learns more reliable distributions of user behaviour. Wang et al. [27] proposed a Variational Autoencoder-Based Adversarial Multimodal Domain Transfer (VAE-AMDT) method, which aligns features from different modalities into a unified embedding space and integrates them through a multi-attention mechanism. In addition, Ye et al. [28] proposed a Cross-Domain Variational Autoencoder (CDVAE), which maps hyperspectral features from different scenes to a shared subspace through a dual-stream VAE and utilizes graph regularization to achieve feature alignment, thereby enhancing cross-scene small-sample classification performance.

These studies demonstrate that VAEs possess great potential in transfer learning. There has been one application of VAE in EEG emotion recognition. Wang et al. [27] proposed a multimodal domain-adaptive variational autoencoder (MMDA-VAE). By constructing a multimodal VAE structure with a shared latent space, it effectively preserves the discriminative information in EEG signals. Combined with adversarial learning and cyclic consistency constraints, it aligns the distributions of multimodal EEG data, reducing the distribution differences between cross-domain data. In contrast, our proposed SCVAE-Net uses single-modal EEG data. And we introduce supervised contrastive constraints and distribution alignment mechanisms into the VAE latent space to further enhance the discriminability and domain invariance of the latent representations.

## 3. Method

In electroencephalogram (EEG) emotion recognition, humans vary in brain structure, physiological state, and emotional response patterns. Therefore, the naturally occurring bias in the distribution of collected emotional EEG data makes it challenging to directly apply a trained model to other new individuals. For this problem, numerous studies indicate that domain adaptation can effectively mitigate the distribution bias caused by individual differences during the process of aligning data distributions between source and target domains.

In multi-source domain adaptation, we denote *K* labelled source domains as $D_{s}={\left\{D_{s}^{\left(k\right)}\right\}}_{k=1}^{K}$ and $D_{s}^{\left(k\right)}={\left\{\left(x_{i}^{s_{k}},y_{i}^{s_{k}}\right)\right\}}_{i=1}^{N_{s_{k}}}∼P_{S_{k}}\left(x,y\right)$, where $x_{i}^{s}$ is the features of the *i*-th sample in the *k*-th source domain with label $y_{i}^{s}$, $N_{s_{k}}$ is the number of source domain samples, and $P_{S_{k}}\left(x,y\right)$ is the distribution of the source domain. In the target domain, we denote the unlabeled target domain as $D_{t}={\left\{x_{j}^{t}\right\}}_{j=1}^{N_{t}}∼P_{T}\left(x\right)$, where $x_{j}^{t}$ is the features of the *j*-th sample in the target domain, $N_{t}$ is the number of samples, and $P_{T}\left(x\right)$ is the distribution. The purpose of domain adaptation is to make the data distributions $P_{S_{k}}\left(x,y\right)$ and $P_{T}\left(x\right)$ of the source and target domains closer so that models performing well in the source domain also perform well in the target domain.

### 3.1. Approach Overview

**Figure 2:**
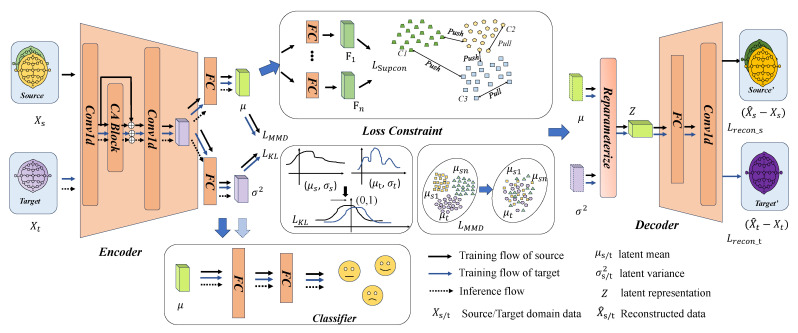
The overall framework of SCVAE-Net. Firstly, the input data is processed by a VAE-based model to extract stable and transferable consistent features across multiple source domains and the target domain. Next, MMD loss is used to align the feature distributions, while KL divergence encourages both to tend towards Gaussian distributions. Finally, multi-view supervised contrastive constraints are applied to the source domain data to enhance the intra-class compactness and inter-class separability of the latent space.

For domain-adaption EEG-based emotion recognition, we propose the Supervised Contrastive Variational AutoEncoder Network (SCVAE-Net). As shown in Figure 2, the overall framework of SCVAE-Net is presented. This model utilizes the probabilistic modeling characteristics of variational autoencoder (VAE) to extract stable and transferable consistent features between multi-source domains and the target domain. However, relying solely on the representation capability of VAE is insufficient to effectively address the EEG emotion classification task in cross-domain. Therefore, SCVAE-Net uses the maximum mean discrepancy (MMD) loss to reduce the distribution discrepancy between the source and target domains in the latent space. To further ensure the classification performance of SCVAE-Net, we adopt a supervised contrastive learning strategy for multi-source domain data, thereby generating features with sufficient discriminability in the latent space. The following subsections will detail our approach.

### 3.2. Channel-Attention-Based Variational Autoencoder

VAE models data distributions in a probabilistic latent space, enabling the learning of transferable representations for cross-domain adaptation. It consists of three parts: an encoder, a latent variable space, and a decoder. For the input data *X*, the output from encoder $q_{\phi }\left(Z|X\right)$ is no longer a deterministic latent representation but rather a probability distribution p(Z), where ϕ represents the parameter of the encoder. So, the latent space features of VAE exhibit continuity, and the p(Z) distribution is typically assumed to converge towards a Gaussian distribution ($N(\mu ,\sigma^{2})$ =N(0,1)). The decoder $p_{\theta }\left(X|Z\right)$ takes the sampled latent variable *Z* as input, with θ being the parameter of the decoder. The core objective function of VAE can be defined as

(1) $$L=E_{q_{\phi }\left(Z|X\right)}\left[\log p_{\theta }\left(X|Z\right)\right]-\mathrm{KL}\;\left(q_{\phi }\left(Z|X\right)\;∥\;p_{\theta }\left(Z\right)\right)$$

The first term is the reconstruction error, measuring the model’s ability to reconstruct data. The second term is the Kullback–Leibler (KL) divergence regularization term, which constrains the posterior distribution *q* to approximate the predefined prior distribution *p*.

#### 3.2.1. Encoder

Although VAE possesses strong feature extraction capabilities, different channels play varying importance in emotion representation and should be explicitly modeled. Therefore, we design a channel attention block within the encoder, enabling the VAE to focus on important channels, thereby enhancing its feature extraction ability. The specific design of the encoder is as follows.

First, the input data $X\in R^{C\times B}$ are organized with *C* channels and *B* frequency bands. In the SEED and SEED-IV datasets [18,19], *C* denotes 62 channels, and *B* denotes 5 frequency bands. Here, 5 frequency bands [18] refer to δ (1–3 Hz), θ (4–7 Hz), α (8–13 Hz), β (14–30 Hz), and γ (31–50 Hz). For *X*, a one-dimensional convolutional layer expands the *C* channels to $C^{\prime }$, extracting deep features $H_{1}\in R^{C^{\prime }\times B}$. It can be shown as

(2) $$H_{1}=\phi \left(W_{1}*X+b_{1}\right)$$

where ϕ(·) is the ReLU activation function, ∗ is a one-dimensional convolution operation, and $W_{1}$ and $b_{1}$ are learnable parameters for convolutional layers.

**Figure 3:**
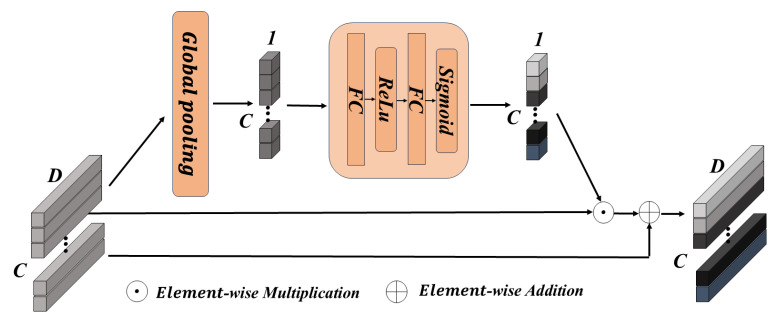
The structure of CA block. The input tensor [C,D] is compressed to [C,1] via global pooling, followed by a nonlinear mapping to generate channel attention weights. These weights are applied to the original features via element-wise multiplication and combined with the input through a residual connection to produce the final output.

Then, we utilize the channel attention block, the structure of which is illustrated in Figure 3. This block compresses feature dimension information through a global average pooling (GAP) layer to extract global features for each channel. Next, a bottleneck structure comprising two fully connected layers performs a nonlinear transformation, using the Sigmoid function to map the learned channel importance weights to (0, 1). These weights are used to perform channel-level weighting on the original features, enabling the VAE to focus on significant channels. After applying a residual connection, we add the weighted features to the original features $H_{1}\in R^{C^{\prime }\times B}$, resulting in the updated features $H_{1}^{\prime }\in R^{C^{\prime }\times B}$. The entire block can be represented by the following formula:

(3) $$H_{1}^{\prime }=H_{1}⊙\sigma \;\left(W_{3}\;\phi \;\left(W_{2}\;\mathrm{GAP}\left(H_{1}\right)+b_{2}\right)+b_{3}\right)+H_{1}$$

where σ(·) is the Sigmoid function; ⊙ denotes element-wise multiplication; and $W_{2}$, $W_{3}$, $b_{2}$ and $b_{3}$ are learnable parameters of the fully connected layers, respectively.

To further extract deeper features, we add a one-dimensional convolutional layer and flatten its output to $H_{2}\in R^{D}$, represented by the following formula:

(4) $$H_{2}=\phi \;\left(R\;\left(W_{4}*H_{1}^{\prime }+b_{4}\right)\right)$$

where R(·) indicates a reshape operation, and $W_{4}$ and $b_{4}$ are learnable parameters for the convolutional layers.

Finally, $H_{2}$ is input into two parallel fully connected layers, which, respectively, output the mean μ and variance $\sigma^{2}$ of the latent distribution, as formulated below:

(5) $$\mu =W_{5}\;H_{2}+b_{5}$$

(6) $$\sigma^{2}=\exp\;\left(W_{6}\;H_{2}+b_{6}\right)$$

where $W_{5}$, $W_{6}$, $b_{5}$, and $b_{6}$ are learnable parameters of the fully connected layers.

The mean μ and variance $\sigma^{2}$ of the latent distribution ensure that the latent space is regular and continuous, providing a reliable foundation for subsequent distribution alignment and discriminative learning. To promote μ and $\sigma^{2}$ to converge towards a Gaussian distribution, KL regularization is employed as a constraint, and it is defined as follows:

(7) $$\begin{array}{cc} L_{\mathrm{KL}}\; & =\mathrm{KL}\;\left(q_{\phi }\left(Z|X\right)\;∥\;p_{\theta }\left(Z\right)\right)\\ \; & =\frac{1}{2}\sum_{i=1}^{d}\left(\mu_{i}^{2}+\sigma_{i}^{2}-\log\sigma_{i}^{2}-1\right) \end{array}$$

where *d* represents the dimension of μ and $\sigma^{2}$.

#### 3.2.2. Decoder

For the decoder, there exists a gradient problem: Directly sampling from random variables renders the gradient non-differentiable with respect to model parameters. Therefore, VAE adopts the reparameterization technique to extract the latent variable *Z* from the inferred distribution as follows:

(8) $$Z=\mu +\exp\;\left(\frac{1}{2}\log\sigma^{2}\right)\cdot \epsilon ,\;\epsilon ∼N\left(0,1\right)$$

where ϵ represents the random noise sampled from a standard normal distribution.

After, the decoder first maps the latent variable *Z* through a fully connected layer and then restores the feature dimension using a reshape operation, followed by feature reconstruction through a one-dimensional convolutional layer to get the reconstructed output $X_{\mathrm{recon}}$. The entire decoder can be represented by the following formula:

(9) $$X_{\mathrm{recon}}=D\left(Z\right)=W_{8}*R\;\left(W_{7}\;Z+b_{7}\right)+b_{8}$$

where D(·) is the decoder function, and $W_{7}$ and $b_{7}$ are learnable parameters for fully connected layers, while $W_{8}$ and $b_{8}$ are learnable parameters for convolutional layers.

We use the L1 norm to calculate the reconstruction loss, promoting the reconstructed $X_{\mathrm{recon}}$ to closely resemble the original input *X* of the VAE, which is defined as follows:

(10) $$\begin{array}{cc} L_{\mathrm{recon}}\; & =E_{q_{\phi }\left(Z|X\right)}\left[\log p_{\theta }\left(X|Z\right)\right]\\ \; & ={∥X_{\mathrm{recon}}-X∥}_{1}\\ \; & =\frac{1}{N}\sum_{i=1}^{N}\left|x_{\mathrm{recon}}^{\left(i\right)}-x^{\left(i\right)}\right| \end{array}$$

where $x_{\mathrm{recon}}^{\left(i\right)}$ is the *i*-th element of the reconstructed output $X_{\mathrm{recon}}$, $x^{\left(i\right)}$ is the *i*-th element of the sample *X*, *N* is the total number of elements in the sample, and ${∥\cdot ∥}_{1}$ is the L1 norm.

### 3.3. Source and Target Domain Alignment

Although the aforementioned VAE can effectively learn consistent representations of EEG data, its domain generalization capability can be further enhanced when facing cross-domain. Therefore, we introduce the MMD [29] method for domain alignment, which brings the feature distributions of the source and target domains closer, allowing the model to learn a more stable representation for domain changes and learn cross-individual features with stronger generalization capabilities. MMD maps samples to a high-dimensional space and compares the mean representations of two domains in this space. It serves as a statistical measure for quantifying the divergence between two distributions. Here, the MMD is used to minimise the difference between the mean $\mu_{s}$ and $\mu_{t}$ of the latent distributions of data from multi-source domain $D_{s}^{\left(k\right)}={\left\{\left({\mu_{s}}_{i}^{s_{k}},y_{i}^{s_{k}}\right)\right\}}_{i=1}^{N_{s_{k}}}∼P_{S_{k}}\left(\mu_{s},y\right)$ and target domain $D_{t}={\left\{{\mu_{t}}_{j}^{t}\right\}}_{j=1}^{N_{t}}∼P_{T}\left(\mu_{t}\right)$. Therefore, the specific formula for MMD can be expressed as follows:

(11) $$\begin{array}{cc} L_{\mathrm{mmd}}\; & ={\mathrm{MMD}}^{2}\;\left(P_{S_{k}}\left(\mu_{s},y\right),\;P_{T}\left(\mu_{t}\right)\right)\\ \; & ={\left|E_{\mu_{s}∼P_{S_{k}}\left(\mu_{s},y\right)}\left[\Phi (\mu_{s})\right]-E_{\mu_{t}∼P_{T}\left(\mu_{t}\right)}\left[\Phi (\mu_{t})\right]\right|}^{2} \end{array}$$

where Φ(·) denotes the kernel mapping function that maps to the reproducing kernel Hilbert space (RKHS), $E_{\mu_{s}∼P_{S_{k}}\left(\mu_{s},y\right)}$ represents the expectation over the distribution $P_{S_{k}}\left(\mu_{s},y\right)$, and $E_{\mu_{t}∼P_{T}\left(\mu_{t}\right)}$ represents the expectation over the distribution $P_{T}\left(\mu_{t}\right)$. However, it cannot be directly calculated, so by extending it to the sample level, it can be expanded as follows:

(12) $$\begin{array}{cc} L_{\mathrm{mmd}}\; & ={\mathrm{MMD}}^{2}\\ \; & =\frac{1}{n_{s}\left(n_{s}-1\right)}\sum_{s\neq s^{\prime }}k\left(\mu_{s},\mu_{s^{\prime }}\right)\\ \; & \;+\frac{1}{n_{t}\left(n_{t}-1\right)}\sum_{t\neq t^{\prime }}k\left(\mu_{t},\mu_{t^{\prime }}\right)\\ \; & \;-\frac{2}{n_{s}n_{t}}\sum_{s=1}^{n_{s}}\sum_{t=1}^{n_{t}}k\left(\mu_{s},\mu_{t}\right) \end{array}$$

where $n_{s}$ and $n_{t}$ represent the number of samples in the source domain and the target domain, respectively, while $\mu_{s}$ and $\mu_{s^{\prime }}$ represent the two latent distribution means in the source domain, while $\mu_{t}$ and $\mu_{t^{\prime }}$ represent the two latent distribution means in the target domain. k(x,y) is a kernel function, typically a Gaussian kernel, as shown below:

(13) $$k\left(x,y\right)=\exp\;\left(-\frac{{∥x-y∥}^{2}}{2a^{2}}\right)$$

where *a* represents the kernel bandwidth, and this kernel function can characterize the similarity between samples. Therefore, the first term of the MMD expansion can be the average similarity within the source domain samples, the second term can be the average similarity within the target domain samples, and the third term can be the average similarity between samples from the source and target domains.

It is worth noting that we did not use the latent variable *Z* because the random sampling noise it introduced by them would fluctuate, leading to unstable distribution estimation and increased gradient noise. μ is equivalent to the core structure of the aligned posterior distribution, enabling more reliable distribution matching. Therefore, SCVAE-Net utilizes MMD to align the data distributions of the target and source domains based on the output latent mean μ of the VAE.

### 3.4. Supervised Multi-View Contrastive Constraint

Although the VAE can extract consistent latent features and the domain alignment methods can reduce distributional differences, they cannot guarantee the model’s ability to distinguish between categories. Therefore, we introduce the supervised multi-view contrastive loss method proposed by Khosla et al. [30] to ensure the classification ability of SCVAE-Net at the label category level.

For the mean $\mu_{i}$ of the latent distribution of labeled sample *i* in the source domain, the *v*-th perspective features $F_{i}^{\left(v\right)}$, extracted by a specially designed linear transformation layer, which is used to construct positive sample pairs, and it can be expressed as follows:

(14) $$F_{i}^{\left(v\right)}=\phi \;\left(W^{\left(v\right)}\;\mu_{i}+b^{\left(v\right)}\right)$$

where $W^{\left(v\right)}$ and $b^{\left(v\right)}$ are the learnable parameters of the fully connected layer in the *v*-th perspective.

For the anchor $F_{i}$, the positive sample set $P\left(i\right)=\left\{F_{p}^{\left(v\right)}|p\neq i,\;y_{p}=y_{i}\right\}\cup \left\{F_{i}^{\left(v\right)}|v=1,2,3,\ldots ,v_{n}\right\}$ is composed of all samples with the same label $y_{i}$, where $v_{n}$ is the number of views, as well as the multi-view features extracted from $F_{i}$ itself through the linear transformation layer. Samples with labels different from $F_{i}$ constitute the negative sample set $M\left(i\right)=\left\{F_{m}^{\left(v\right)}|y_{m}\neq y_{i}\right\}$. By maximizing the similarity between the anchor $F_{i}$ and the positive sample $F_{p}$ from any perspective while minimizing the similarity between $F_{i}$ and the negative sample $F_{m}$ from any viewpoint, the model promotes intra-class compactness and inter-class separability in the feature space. The entire supervised multi-view contrastive loss can be expressed as follows:

(15) $$L_{\sup}=\sum_{i=1}^{N}\frac{-1}{\left|P(i)\right|}\sum_{p\in P(i)}\log\frac{\exp\;\left(\mathrm{sim}(F_{i},F_{p})/\tau \right)}{\sum_{m\in M(i)}\exp\;\left(\mathrm{sim}(F_{i},F_{m})/\tau \right)}$$

where *N* is the total number of samples, and τ represents the temperature coefficient, which adjusts the smoothness of the similarity distribution. In our study, sim(x,y) employs cosine similarity:

(16) $$\mathrm{sim}\left(x,y\right)=\frac{x^{⊤}\cdot y}{∥x∥\;∥y∥}$$

where ∥·∥ represents the Euclidean norm.

### 3.5. All Loss Functions

In summary, SCVAE-Net primarily comprises three main steps. Firstly, input the source and target domains data into a shared VAE to obtain the reconstructed output $X_{\mathrm{recon}}$, the mean μ and variance $\sigma^{2}$ of the latent distribution, and the resampled latent variable *Z*, where the reconstruction loss is computed using the L1 norm. Then, align all source and target domains to learn high-level domain-invariant features by minimising the MMD. Next, through multi-view supervised contrastive loss, narrow the distribution of the same category and push away the distribution between different categories, further constraining the consistency of features across different views within the same category. Finally, we use a classifier with two fully connected layers to classify the extracted features μ, as shown in Figure 2, using cross-entropy loss as the classification loss:

(17) $$L_{\mathrm{cla}}=-\frac{1}{N}\sum_{i=1}^{N}\sum_{c=1}^{C}y_{i,c}\log\left(p_{i,c}\right)$$

where *N* is the total number of samples, *C* is the number of categories, $y_{i,c}$ is the true label of the ith sample, and $p_{i,c}$ is the predicted probability of the *i*-th sample belonging to category C. For the SEED dataset, C = 3, representing the emotional categories positive, negative, and neutral, whereas for SEED-IV, C = 4, representing happy, sad, fear, and neutral.

All in all, the calculation of total loss is as follows:

(18) $$L_{\mathrm{total}}=L_{\mathrm{cla}}+\lambda_{1}L_{\mathrm{recon}}+\lambda_{2}L_{\sup}+\lambda_{3}L_{\mathrm{mmd}}+\lambda_{4}L_{\mathrm{kl}}$$

where $\lambda_{1},\;\lambda_{2},\;\lambda_{3},\;and\;\lambda_{4}$ represent the weight parameters for each loss, respectively.

### 3.6. Experiments

#### 3.6.1. Datasets and Experimental Protocol

SEED [18] and SEED-IV [19] are widely used benchmark datasets for EEG-based emotion recognition. Both datasets include EEG recordings from 15 participants, each completing three experimental sessions, and employ a 62-channel EEG acquisition system with a sampling rate of 1000 Hz. In each session, participants watched a series of film clips designed to elicit emotions, during which EEG signals were recorded. Specifically, SEED contains 15 clips per session, including 5 clips for each emotion, and each clip lasts approximately 4 min. The dataset adopts a three-class emotion labeling scheme consisting of sad, neutral, and happy. In contrast, SEED-IV includes 24 clips per session, with 6 clips assigned to each emotion category, and each clip lasts either 2 min or 4 min. The dataset uses a four-class emotion labeling scheme consisting of happiness, sadness, fear, and neutrality, which makes the emotion discrimination task more challenging than that in SEED.

For the raw EEG data, we perform preprocessing operations according to the procedures in [18]. First, the raw EEG signals are downsampled to 200 Hz. Second, we apply a 50 Hz notch filter to suppress power interference. Next, band-pass filtering is applied using a 4th-order Butterworth filter (0–75 Hz). Then, the signals are decomposed into 5 frequency bands: δ, θ, α, β and γ. Finally, the preprocessed signals are segmented into non-overlapping 4-s windows. We use differential entropy (DE) to perform feature extraction of EEG data in these 5 frequency bands. The DE features can be represented by the following formula [31]:

(19) $$\begin{array}{cc} DE=h(x)\; & =-\int_{-\infty }^{\infty }\frac{1}{\sqrt{2\pi \sigma^{2}}}\exp\frac{{\left(x-\mu \right)}^{2}}{2\sigma^{2}}\ln\left(\frac{1}{\sqrt{2\pi \sigma^{2}}}\exp\frac{{\left(x-\mu \right)}^{2}}{2\sigma^{2}}\right)dx\\ \; & =\frac{1}{2}\ln2\pi e\sigma^{2} \end{array}$$

where *x* represents the processed waveform, x∼N(0,1); μ denotes the average amplitude of the EEG signal; and $\sigma^{2}$ is the variance of the signal.

To evaluate the classification performance of the model, we conducted two types of experiments on the SEED and SEED-IV datasets: (1) Cross-Subject: For cross-subject emotion recognition, the leave-one-subject-out protocol was adopted. In each iteration, one subject was regarded as the target domain and held out for testing, while the remaining subjects were treated as source domains and used for model training. The procedure was repeated for all subjects, and the final performance was obtained by averaging the results over all LOSO iterations. (2) Cross-Session: For cross-session emotion recognition, a leave-one-session-out protocol was adopted. In each iteration, the EEG data from one session were held out for testing, while the data from the remaining two sessions were used for training. Unlike the cross-subject setting, the subjects in the training and test sets were the same, and the domain shift mainly came from different recording sessions. The procedure was repeated until each of the three sessions had been used once as the test session, and the average performance across the three iterations was reported for each dataset.

#### 3.6.2. Implementation Details

The input EEG data for SCVAE-Net is $X\in R^{C\times B}$, where C denotes the number of channels and is 62 in the SEED and SEE-IV datasets, and B is 5. The output dimensions of intermediate features (such as μ and $\sigma^{2}$) in SCVAE-Net are set to 128. In the constraint of multi-view supervised contrastive loss, the number of views is set to 2, and the temperature coefficient τ is empirically set to 0.1. In terms of hardware, we conduct model training and testing on a laptop equipped with an AMD Radeon 780M Graphics GPU. Additionally, we utilize the PyTorch framework (version 2.3.1 + cu121, with CUDA 12.1 support), select Adam as the optimizer, and adjust the learning rate within the range of 0.002 to 0.003. In addition, considering training efficiency and experimental consistency, all experimental training epochs are uniformly set to 300, and the batch size is set to 128. More parameters, such as the weight parameters of various losses $\left(\lambda_{1},\lambda_{2},\lambda_{3}\;\mathrm{and}\;\lambda_{4}\right)$, the number of viewpoints, and the intermediate feature dimension, will be explored in the subsequent parameter sensitivity analysis. The relevant code is available at https://github.com/braverSheep/SCVAE-Net (accessed on 13 May 2026).

### 3.7. Evaluation Metrics and Statistical Analysis

To comprehensively evaluate the classification performance of SCVAE-Net, we adopted four commonly used metrics, including accuracy (Acc), F1 score, precision, and recall. Accuracy reflects the overall proportion of correctly classified samples. Since both SEED and SEED-IV are multi-class emotion recognition datasets, precision, recall, and F1 score were calculated in a macro-averaged manner, i.e., the metric was first computed for each emotion category and then averaged across all categories.

In addition, when detailed paired experimental results were available, we further conducted statistical significance analysis to assess whether the performance differences between SCVAE-Net and the comparison variants were significant. Specifically, for each comparison, the results of 15 subjects across all three sessions were jointly used to form 45 paired subject/session-level values. The paired differences between SCVAE-Net and each comparison variant were then calculated under the same subject and session. Since these paired differences did not satisfy the normality assumption required by the paired *t*-test, the Wilcoxon signed-rank test was adopted to calculate the *p*-values. The significance level was set to α=0.05, and ^*^, ^**^, and ^***^ denote p<0.05, p<0.01, and p<0.001, respectively.

## 4. Results

### 4.1. Cross-Subject and Cross-Session Results

**Figure 4:**
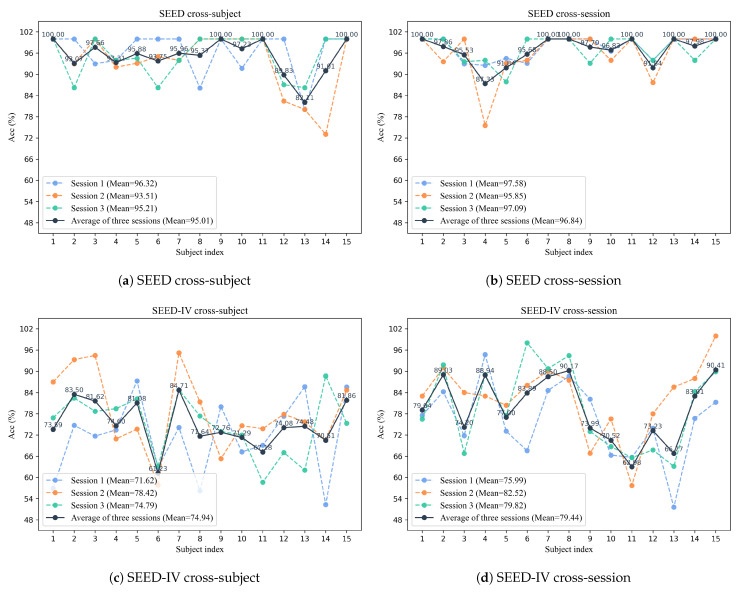
Detailed experimental results on SEED and SEED-IV datasets.

Figure 4 presents the detailed experimental results of the proposed model on the SEED and SEED-IV datasets under both cross-subject and cross-session settings. The figure reports the classification accuracy of each subject across three sessions, as well as the average accuracy over all sessions.

On the SEED dataset, under the cross-subject setting as shown in Figure 4a, the average accuracies of Session 1, Session 2, and Session 3 are 96.32 ± 06.01%, 93.51 ± 08.25%, and 95.21 ± 05.75%, respectively, with an overall average accuracy of 95.01 ± 06.67%. Under the cross-session setting in Figure 4b, the average accuracies of Session 1, Session 2, and Session 3 are 97.58 ± 03.08%, 95.85 ± 06.60%, and 97.09 ± 03.81%, respectively, and the overall average accuracy reaches 96.84 ± 04.49%.

As shown in Figure 4c, the results under the cross-subject setting are illustrated on the SEED-IV dataset, where the average accuracies of Session 1, Session 2, and Session 3 are 71.62 ± 10.62%, 78.42 ± 10.53%, and 74.79 ± 08.51%, respectively, yielding an overall average accuracy of 74.94 ± 09.88%. In comparison, as shown in Figure 4d, the cross-session setting achieves higher accuracies of 75.99 ± 10.38%, 82.52 ± 09.79%, and 79.82 ± 11.54% for the three sessions, respectively, with an overall average accuracy of 79.44 ± 10.57%.

Furthermore, we observe that the cross-session setting consistently achieves higher accuracy than the cross-subject setting on both datasets. For example, on the SEED dataset, the overall accuracy improves from 95.01 ± 06.67% to 96.84 ± 04.49%, and on the SEED-IV dataset, it increases from 74.94 ± 08.51% to 79.44 ± 10.57%.

**Figure 5:**
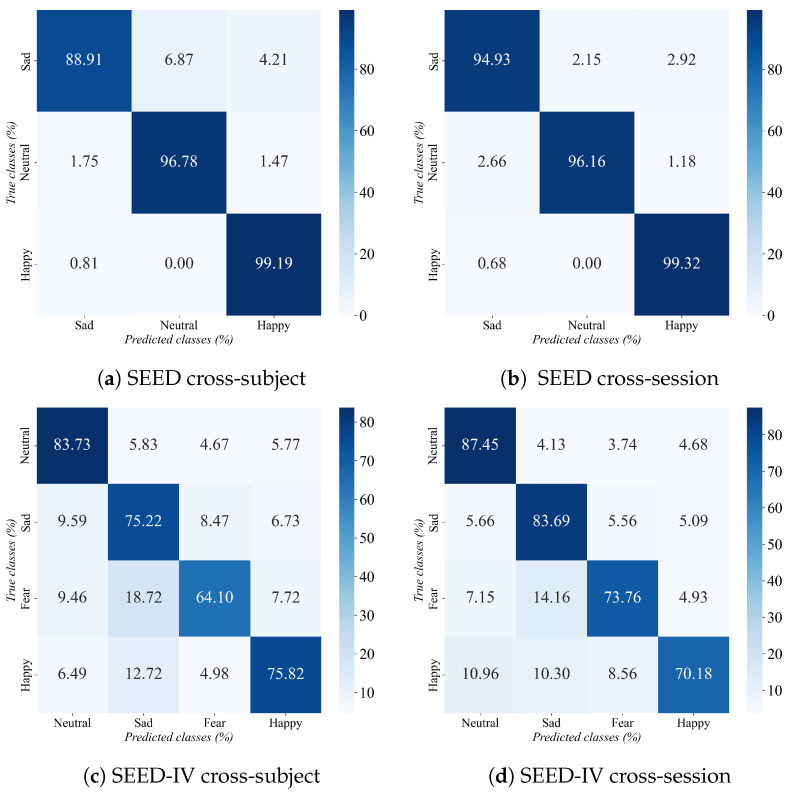
Confusion matrices under cross-subject and cross-session settings on the SEED and SEED-IV datasets.

Figure 5 presents the confusion matrix for SCVAE-Net’s emotional classification on the SEED and SEED-IV datasets. On the SEED dataset, the model demonstrates high classification accuracy under both experimental settings, with the three emotional categories being effectively distinguished. In particular, in cross-session experiments, although the confusion in recognizing the Sad category slightly increases, the overall performance remains stable. In comparison, on the SEED-IV dataset, the model performs more pronounced classification confusion due to the presence of four emotion categories. A degree of mutual confusion exists between the sad and fear categories, while the happy category demonstrates weaker separability and is more prone to misclassification compared to other emotion categories. Overall, classification performance in the cross-session scenario generally outperforms that in the cross-subject scenario.

### 4.2. Comparison with Other Methods

To evaluate the performance of SCVAE-Net in domain adaptation for EEG emotion recognition, we compare it with other methods, as shown in Table 1. From the table, it can be seen that among all the compared methods, SCVAE-Net achieves the best results on both datasets and under both evaluation settings: On the SEED dataset, its cross-subject accuracy reaches 95.01 ± 06.67%, an improvement of 1.26% compared to the second-highest ASJDA [32]. Its cross-session accuracy reaches 96.84 ± 04.49%, an improvement of 0.73% compared to the second-highest $S^{2}A^{2}‑\mathrm{MSDA}$ [33]. On the more challenging SEED-IV dataset, SCVAE-Net achieves 74.94 ± 08.51% and 79.44 ± 10.57% in cross-subject and cross-session settings, respectively, surpassing the second-highest model ADANN [34] by 0.99% and 1.65%.

These results indicate that SCVAE-Net performs greater robustness and generalisation ability in mitigating cross-subject and cross-session distribution variations, validating its performance in EEG emotion recognition tasks. Furthermore, we observe that models generally achieve better performance under the cross-session setting than under the cross-subject setting. This suggests that inter-subject variability tends to be substantially greater.

To provide a more comprehensive comparison of the models, we include comparisons of parameter size, FLOPs, and inference time, as shown in Table 2. Regarding parameter size, SCVAE-Net has 662.21K learnable parameters and 356.35K FLOPs, making it a lightweight to medium-sized network. Its single inference time is approximately 0.000786 s per sample, enabling it to process over 1200 samples per second, which is sufficient for real-time applications. While SCVAE-Net is not the smallest or fastest among all models, its high accuracy demonstrates a strong balance between efficiency and performance.

### 4.3. Ablation Studies

To further understand SCVAE-Net, we conduct ablation experiments to evaluate the impact of the channel attention block, source–target domain alignment, and supervised multi-view contrastive constraints on domain adaptation. We perform ablation experiments on both the SEED and SEED-IV datasets, and the results are presented in Table 3. Here, “w/o $L_{\mathrm{recon}}$” denotes the removal of the reconstruction loss in SCVAE-Net, “w/o $L_{\sup}$” denotes removal of the supervised multi-view contrastive constraint, “w/o $L_{\mathrm{mmd}}$” denotes removal of the source–target domain alignment method, “w/o $L_{\mathrm{kl}}$” denotes removal of KL divergence, and “w/o CA block” denotes removal of the channel attention block in the VAE.

As shown in Table 3, both the channel attention block and the various loss terms within SCVAE-Net play an indispensable role in the model’s performance. On the SEED dataset, the complete model achieves maximum accuracy rates of 95.01 ± 06.67% and 96.84 ± 04.49% under cross-subject and cross-session settings, respectively. When any loss term or channel attention block is removed, the performance decreases to varying degrees. Among them, the performance degradation is most pronounced when $L_{\mathrm{mmd}}$ is removed, with decreases of 11.54% and 7.47% in cross-subject and cross-session settings, respectively. This indicates that the domain alignment mechanism plays an important role in mitigating inter-subject distribution differences and enhancing cross-subject generalization ability. Removing $L_{\sup}$, $L_{\mathrm{kl}}$, $L_{\mathrm{recon}}$, or the CA block also leads to a decrease in accuracy, indicating that the supervised multi-view comparison constraint, latent space regularisation, reconstruction loss, and channel attention block are equally crucial for learning discriminative and generalisable features.

On the SEED-IV dataset, the complete SCVAE-Net model achieves optimal results of 74.94 ± 08.51% and 79.44 ± 10.57% under both cross-subject and cross-session settings. After removing the source–target domain alignment loss $L_{\mathrm{mmd}}$, the model’s accuracy significantly decreased by 9.43% and 5.70% on the two datasets, respectively. Compared to SEED, the contributions of KL regularization $L_{\mathrm{kl}}$ and reconstruction loss $L_{\mathrm{recon}}$ are more pronounced on SEED-IV. Although the supervised contrastive loss and channel attention block have a smaller contribution, they also enhance the model. In summary, the ablation experiments validate the synergistic interaction among the constituent modules of SCVAE-Net, demonstrating that the reconstruction constraints, supervisory information, domain alignment mechanism, and channel attention block collectively enhance the model’s robustness and generalisation performance in EEG emotion recognition tasks across both cross-subject and cross-session settings.

### 4.4. Source Subject Numbers

Due to the substantial inter-subject variability in EEG signals, the number of source domains may affect the ability of a model to learn subject-invariant and transferable representations. Therefore, we further investigate how the number of source domains influences the cross-subject recognition performance of SCVAE-Net.

**Figure 6:**
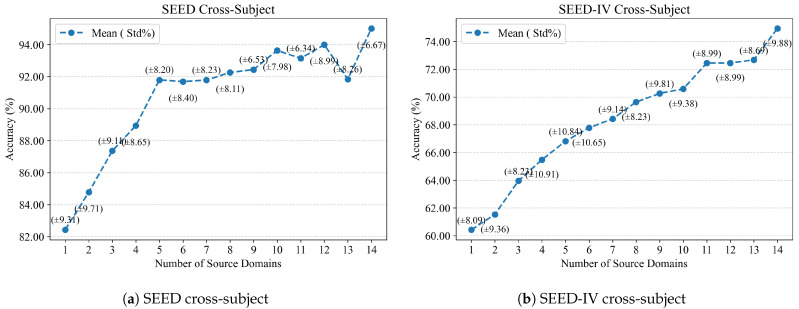
Performance comparison under different numbers of source domains on the SEED and SEED-IV datasets.

In these experiments, we also adopt the leave-one-subject-out cross-validation with circular source domain selection, inspired by the concept of k-fold cross-validation. Specifically, for a given number of source domains *n*, one subject is selected as the target domain at a time. The source domains are chosen consecutively from the subjects preceding the target domain, and if the number of available subjects is insufficient, the selection wraps around from the end of the sequence to maintain a constant number of source domains. For example, when the number of source domains is three and the target domain is subject 2, the source domains are subjects 14, 15, and 1; when the number of source domains is two and the target domain is subject 3, the source domains are subjects 1 and 2. The average classification accuracy and standard deviation across all target domains are calculated and shown in Figure 6, reflecting the robustness of the results.

As shown in Figure 6, in the cross-subject setting of the SEED and SEED-IV datasets, the model’s performance generally shows an upward trend with an increase in the number of source domains. When the number of source domains is low, the model’s performance is suboptimal, indicating that a single or small number of source domains is difficult to adequately characterize the representative common features in EEG. As the number of source domains gradually increases, the model is able to learn richer and more stable cross-subject discriminative information from more subjects, leading to a gradual improvement in classification accuracy. On the SEED dataset, when the number of source domains reaches a medium scale, the performance improvement gradually slows down, and small fluctuations occur with a larger number of source domains. On the SEED-IV dataset, the performance improvement trend is more sustained, indicating that multi-source domain information has a more important promoting effect on complex emotional classification tasks. This experiment validates that increasing the number of source domains can effectively enhance the model’s cross-subject generalization ability. These results suggest that SCVAE-Net can effectively extract richer cross-subject discriminative information from multiple source domains, enabling accurate predictions and achieving better results in the target domain.

### 4.5. Parameter Sensitivity Analysis

To evaluate the impact of variations in key hyperparameters on the SCVAE-Net, we conduct parameter sensitivity experiments on the SEED dataset. These experiments focus on the weighting parameters $\lambda_{1},\;\lambda_{2},\;\lambda_{3},\;\mathrm{and}\;\lambda_{4}$ (corresponding to the losses $L_{\mathrm{recon}}$, $L_{\sup}$, $L_{\mathrm{mmd}}$ and $L_{\mathrm{kl}}$), as well as the number of viewpoints and the dimensionality of the feature extractor.

**Figure 7:**
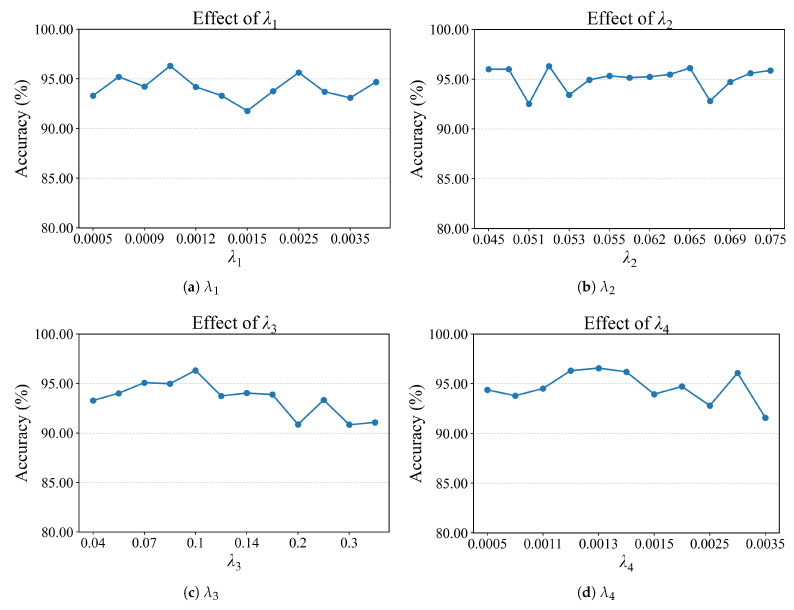
Parameter sensitivity analysis on the SEED dataset under the cross-subject setting.

The effect of the weight parameters for each loss function ($\lambda_{1},\;\lambda_{2},\;\lambda_{3},\;\lambda_{4}$) on the classification accuracy of SCVAE-Net is presented in Figure 7. As shown in the results, the variations of $\lambda_{1}$, $\lambda_{2}$, and $\lambda_{4}$ have a relatively limited influence on performance, where accuracy remains stable within approximately 92–96.5%, indicating good robustness of the model to these parameters. In particular, $\lambda_{2}$ shows only minor fluctuations around 95%–96%, except for a slight drop to about 92.5% at 0.065–0.070. In comparison, $\lambda_{3}$ has a more significant impact on performance. The accuracy reaches its peak of about 96% when $\lambda_{3}$ is around 0.10–0.12, while increasing $\lambda_{3}$ to larger values leads to a clear decrease to around 90%–91%, demonstrating the sensitivity of the model to the domain alignment term. These quantitative results once again highlight the importance of the domain alignment mechanism for SCVAE-Net.

Table 4 presents the experimental results for supervised contrastive learning under different settings for the number of viewpoints. From this table, it can be seen that SCVAE-Net achieves the best performance when using two viewpoints, with accuracies of 96.31 ± 06.01%, 93.50 ± 08.25%, and 95.21 ± 05.75% for Session 1, Session 2, and Session 3, respectively, with an average accuracy of 95.01 ± 06.67%. This indicates that introducing multi-perspective information appropriately can characterize sample features from different perspectives, thereby providing more comprehensive emotion-related information and enhancing the model’s ability to express emotions. In contrast, when using only a single viewpoint, the average accuracy of the model is 93.23 ± 07.09%, indicating that a single viewpoint contains relatively limited information and is difficult to fully characterize sample features. When the number of viewpoints increases to four and six, the average accuracy of the model is 92.79 ± 06.77% and 93.64 ± 06.06%, respectively, indicating that too many viewpoints may bring redundant information and increase the complexity of feature fusion. Although the model achieves a maximum accuracy of 95.79 ± 04.86% on Session 3 using six viewpoints, its overall average accuracy is still lower than that of the model using two viewpoints.

The experimental results for the feature extractor dimension before the input contrastive learning module are shown in Table 5. From this table, it can be seen that when the dimension is set to 128, the model achieves the highest accuracy, indicating that 128 dimensional features can better retain emotion-related information and thus learn more discriminative emotion feature representations. By contrast, when the dimensions are low, such as 16, 32, and 64, although the model can also achieve good results, the average accuracy is lower than 128 dimensions, indicating that the feature representation ability of lower dimensions is limited, and it is difficult to fully characterize the emotional information of the sample. However, when the dimension increases to 256 and 512, model performance decreases somewhat, indicating that excessively high dimension may introduce redundant features, thereby affecting the model’s generalisation ability. Although the 512 dimensions achieve the highest accuracy of 95.50 ± 05.68% on Session 3, its overall average performance is still not as stable as 128 dimensions.

### 4.6. Input of Latent Features

In VAE, latent representations typically include mean μ, variance $\sigma^{2}$, and random sampling variable *Z*. Different forms of latent features may differ in terms of stability, discriminability, and robustness. In existing research, some methods use only μ as the classification representation [45,46], while others construct latent representations by combining μ and $\sigma^{2}$ [47,48].

Therefore, to analyze the impact of different latent feature combinations on SCVAE-Net, we conduct experiments on the SEED dataset using two sets of different latent feature combinations: (1) Under the condition of keeping the input of other modules unchanged, we change only the latent representation inputs to the classifier, and the results are shown in Table 6. (2) Furthermore, considering that the role of latent representation may be affected by factors such as reconstruction loss, contrastive loss, and MMD domain alignment, based on Experiment (1), the latent representation inputs of the classifier and each loss function are simultaneously changed. The results are shown in Table 7. Through these two sets of experiments, the impact of different latent representations on model performance can be analyzed from both single-factor and multi-factor perspectives, thereby verifying whether the selected latent representations’ performance exhibits good stability.

Table 6 shows the accuracy of SCVAE-Net when only the latent representation of the classifier input is changed. From this table, it can be seen that when only the latent mean μ is used as the classifier input, the model achieves optimal performance, with accuracies of 95.01 ± 06.67% and 96.84 ± 04.49% under cross-subject and cross-session settings, respectively. This indicates that the latent mean μ can more stably and effectively represent the discriminative information of the sample. In contrast, the performance of the model decreases more significantly when using only $\sigma^{2}$, indicating that variance focuses more on characterizing the uncertainty of the distribution and has difficulty providing sufficient discriminative power when used directly for classification. When adding or concatenating μ and $\sigma^{2}$ and inputting them into the classifier, although the model’s performance has improved compared to using only $\sigma^{2}$, the overall result is still not as good as using only μ, indicating that introducing variance may interfere with the original clear discriminative representation.

Table 7 shows the impact of different combinations of latent representations on model performance when the reconstruction loss, contrastive loss, MMD domain alignment, and classifier input latent features are simultaneously changed. It can be seen that when only using μ as the latent feature, the model achieves the best results in both cross-subject and cross-session, with accuracies of 95.01 ± 06.67% and 96.84 ± 04.49%, respectively. This once again demonstrates that μ can stably retain discriminative emotional information and has stronger robustness. In contrast, the performance of SCVAE-Net decreases when using $\sigma^{2}$ and adding or concatenating μ and $\sigma^{2}$, further illustrating that variance tends to reflect the uncertainty of the underlying distribution. Table 6 explores the impact of changing only the input latent features of the classifier on model performance while keeping other conditions constant. Differently, Table 7 presents experiments on different latent feature combination schemes with varying loss functions and classifier inputs. However, both experimental results indicate that the performance is best when using only μ, which further demonstrates that μ has good stability and reliability under different settings.

### 4.7. Visualization of Results

**Figure 8:**
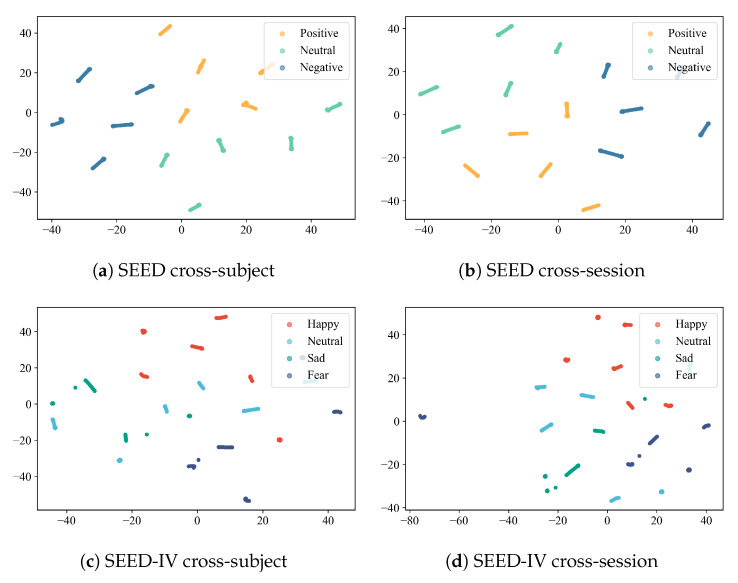
Visualization results under cross-session and cross-subject settings on the SEED and SEED-IV datasets for Subject 1 in Session 1.

We utilize t-SNE for visualisation across both cross-subject and cross-session settings within the SEED and SEED-IV datasets, as shown in Figure 8. For convenient presentation, we uniformly select data from subject 1 in Session 1 across both datasets for visualisation. In the SEED dataset, different emotion categories have formed relatively clear clustering structures under two settings. The distribution of samples within each class is relatively compact, and the gap between classes is evident, indicating that the model can effectively learn discriminative emotion-related features. In the cross-session scenarios, the feature distribution maintains good separability, indicating that the model exhibits good robustness to session variations. In contrast, the SEED-IV dataset shows greater dispersion across its overall distribution due to its expanded range of emotional categories and heightened task difficulty. Nevertheless, fundamental distinctions between emotional categories within the latent space remain observable under both experimental settings. The visualisation results validate that SCVAE-Net can learn clear and discriminative latent representations under cross-subject and cross-session conditions, further demonstrating the proposed method’s robust generalisation capability and stability in complex cross-domain EEG emotion recognition tasks.

### 4.8. Limitations

The proposed SCVAE-Net shows promising performance. However, several limitations remain. First, as shown in Table 2, SCVAE-Net has a total of 662.21K parameters, which is much larger than those of other methods, indicating higher computational cost and potential overfitting risk. Second, the experimental results show that SCVAE-Net can achieve over 90% accuracy on the SEED dataset, but its performance on SEED-IV is much lower, indicating that the model’s recognition ability needs further improvement on more complex datasets.

To address these limitations, we plan to use network compression and regularization techniques to reduce parameter size and alleviate overfitting. Additionally, we will adopt data augmentation and multi-view feature fusion strategies to enhance the model’s generalization ability on more complex datasets.

## 5. Conclusions

Cross-domain EEG emotion recognition is a challenging task, primarily focused on reducing distributional differences between target and source domains. Based on this, we propose a supervised contrastive VAE network (SCVAE-Net), which utilizes the reconstruction mechanism and probabilistic nature of the latent space in VAE to enhance its ability to extract consistent features across multiple domains. To mitigate the degradation of discriminative capabilities during domain alignment, we introduce a multi-view supervised contrastive loss to enhance the intra-class consistency and inter-class separability of the intermediate features output by the VAE. We conducted cross-subject and cross-session experiments on the SEED and SEED-IV datasets. The results demonstrate that the VAE-based SCVAE-Net achieves satisfactory performance, validating the effectiveness of the proposed method.

In the future, we will further explore cross-domain latent space modeling and distribution alignment strategies to enhance the model’s adaptability in complex cross-subject and cross-session scenarios. Simultaneously, we will attempt to extend SCVAE-Net to emotion recognition tasks in multimodal or weakly supervised settings.

## Figures and Tables

**Table 1:** Classification accuracy (%) comparison under cross-subject and cross-session settings on SEED and SEED-IV datasets.

Dataset	Methods	Cross-Subject	Cross-Session
SEED	BiHDM [35]	85.40±07.53	–
	DDA [36]	91.08±07.70	92.89±07.64
	PR-RL [37]	85.56±04.78	93.18±06.55
	GCD-JFSE [38]	78.67±14.67	87.11±10.58
	FMLAN [39]	90.96±07.05	91.94±07.73
	GDDN [40]	92.54±03.65	–
	MMDA-VAE [27]	85.07±11.81	–
	ADANN [34]	91.92±05.44	90.03±06.99
	$S^{2}A^{2}‑\mathrm{MSDA}$ [33]	90.11±07.32	96.11±05.01
	LGDAAN-Nets [41]	89.09±07.76	91.25±05.87
	ASJDA [32]	$\underline{93.75\pm 11.39}$	–
	**SCVAE-Net (Ours)**	95.01±06.67	96.84±04.49
SEED-IV	GCD-JFSE [38]	58.98±10.62	60.74±11.93
	BiDANN [10]	65.59±10.39	–
	BiHDM [35]	69.03±08.66	–
	DDA [36]	73.14±09.43	75.96±11.55
	MSDA-SFE [42]	73.92±06.04	76.16±11.77
	MASTF-net [43]	73.51±06.89	–
	${\mathrm{HVF}}_{2}N‑\mathrm{DBR}$ [44]	73.60±02.91	–
	ADANN [34]	$\underline{73.95\pm 09.08}$	$\underline{77.79\pm 13.97}$
	**SCVAE-Net (Ours)**	74.94±09.88	79.44±10.57

The best results are highlighted in bold, and the second-best results are underlined.

**Table 2:** Comparison of model complexity, inference speed, and accuracy on the SEED dataset.

Model	Params	FLOPs	Inference Time (s)	Cross-Subject (%)	Cross-Session (%)
DDA	251.52 K	250.69 K	0.000545	91.08±07.70	92.89±07.64
PR-RL	96.26 K	95.94 K	0.000171	85.56±04.78	93.18±06.55
$S^{2}A^{2}‑\mathrm{MSDA}$	159.20 K	2.6 M	0.003687	90.11±07.32	96.11±05.01
ASJDA	154.41 K	153.44 K	0.003206	93.75±11.39	–
SCVAE-Net (Ours)	662.21 K	356.35 K	0.000786	95.01±06.67	96.84±04.49

**Table 3:** Ablation study of SEED and SEED-IV with SCVAE-Net.

Dataset	Methods	Cross-Subject				Cross-Session			
		Acc ± Std (%)	F1 (%)	Precision (%)	Recall (%)	Acc ± Std (%)	F1 (%)	Precision (%)	Recall (%)
SEED	SCVAE-Net	**95.01 ± 06.67**	**94.65**	**96.06**	**94.95**	**96.84 ± 04.49**	**96.74**	**97.46**	**96.80**
	w/o $L_{\mathrm{recon}}$	93.17 * ± 06.94	92.93	93.98	93.10	96.75 ± 05.16	96.65	97.12	96.71
	w/o $L_{\sup}$	92.56 *** ± 06.94	92.02	94.03	92.46	94.35 ** ± 06.16	94.16	95.27	94.29
	w/o $L_{\mathrm{mmd}}$	83.47 *** ± 09.75	82.62	85.85	83.35	89.37 *** ± 08.34	88.83	91.28	89.31
	w/o $L_{\mathrm{kl}}$	92.82 * ± 06.79	92.59	93.59	92.72	94.86 *** ± 06.12	94.75	95.40	94.80
	w/o CA block	93.23 * ± 06.08	92.98	94.29	93.15	95.01 ** ± 06.04	94.72	95.88	94.94
SEED-IV	SCVAE-Net	**74.94 ± 09.88**	**72.88**	**76.88**	**73.89**	**79.44 ± 10.57**	77.57	80.63	78.57
	w/o $L_{\mathrm{recon}}$	70.85 *** ± 09.63	67.87	70.49	69.75	77.27 ** ± 11.05	75.51	78.20	76.68
	w/o $L_{\sup}$	71.72 *** ± 09.65	68.78	72.57	70.56	79.05 ± 10.61	**78.12**	**80.88**	**78.77**
	w/o $L_{\mathrm{mmd}}$	65.51 *** ± 09.69	59.86	61.51	64.27	73.74 *** ± 11.12	71.70	75.73	72.86
	w/o $L_{\mathrm{kl}}$	71.34 *** ± 09.24	68.59	71.85	70.42	76.80 *** ± 11.80	75.10	77.22	76.27
	w/o CA block	73.18 ** ± 10.91	69.36	71.73	71.99	77.51 * ± 11.08	75.58	78.69	77.19

The best results are highlighted in bold. Statistical comparisons were conducted between each variant and the full SCVAE-Net under the same experimental setting. For each comparison, the results from three sessions and 15 subjects were pooled as paired subject–session observations. The Wilcoxon signed-rank test was used to compute the *p*-values. *, **, and *** indicate statistical significance at p<0.05, p<0.01, and p<0.001, respectively.

**Table 4:** Performance of different views on three sessions of SEED dataset.

View	Session 1	Session 2	Session 3	Average			
				Acc ± Std (%)	F1 (%)	Precision (%)	Recall (%)
1	95.75 ± 04.19	88.64 ± 11.23	95.30 ± 05.78	93.23 ** ± 07.09	92.84	94.80	93.16
2	**96.31 ± 06.01**	**93.50 ± 08.25**	95.21 ± 05.75	**95.01 ± 06.67**	**94.65**	**96.06**	**94.95**
4	95.81 ± 03.57	88.89 ± 11.20	93.69 ± 05.55	92.79 *** ± 06.77	91.98	93.91	92.25
6	94.51 ± 05.37	90.64 ± 07.95	**95.79 ± 04.86**	93.64 * ± 06.06	93.40	94.65	93.57

The best results are highlighted in bold. The Wilcoxon signed-rank test was used to compute the *p*-values. *, **, and *** indicate statistical significance at p<0.05, p<0.01, and p<0.001, respectively.

**Table 5:** Performance under different feature extractor dimensions on three sessions of SEED dataset.

Feature Extractor Dimension	Session 1	Session 2	Session 3	Average			
				Acc ± Std (%)	F1 (%)	Precision (%)	Recall (%)
16	94.32 ± 04.96	89.23 ± 09.29	93.49 ± 05.06	92.34 *** ± 06.43	92.07	93.16	92.26
32	94.03 ± 04.98	90.08 ± 08.44	95.08 ± 04.88	93.06 *** ± 06.10	92.80	93.88	92.97
64	93.77 ± 06.55	89.27 ± 07.94	93.82 ± 06.45	92.28 *** ± 06.98	91.91	93.42	92.19
128	**96.31 ± 06.01**	**93.50 ± 08.25**	95.21 ± 05.75	**95.01 ± 06.67**	**94.65**	**96.06**	**94.95**
256	90.19 ± 09.86	89.77 ± 08.51	93.22 ± 06.09	91.06 *** ± 06.67	90.46	92.35	90.96
512	93.05 * ± 05.74	89.45 * ± 07.43	**95.50 ± 05.68**	92.67 ** ± 06.28	92.36	93.93	92.59

The best results are highlighted in bold. The Wilcoxon signed-rank test was used to compute the *p*-values. *, **, and *** indicate statistical significance at p<0.05, p<0.01, and p<0.001, respectively.

**Table 6:** Performance on SEED dataset when only the classifier input is changed.

Classifier Input	Cross-Subject				Cross-Session			
	Acc ± Std (%)	F1 (%)	Precision (%)	Recall (%)	Acc ± Std (%)	F1 (%)	Precision (%)	Recall (%)
μ	**95.01 ± 06.67**	**94.65**	**96.06**	**94.95**	**96.84 ± 04.49**	**96.74**	**97.46**	**96.80**
$\sigma^{2}$	89.11 *** ± 07.65	88.66	91.35	89.01	94.27 ** ± 06.84	94.18	95.11	94.23
$\mu +\sigma^{2}$	89.73 *** ± 07.86	89.23	91.47	89.62	95.53 ± 06.21	95.44	96.21	95.48
$\left[\mu ,\sigma^{2}\right]$	92.22 ** ± 07.51	91.9	93.61	92.13	95.06 * ± 05.62	94.71	96.09	95.00

The best results are highlighted in bold. The Wilcoxon signed-rank test was used to compute the *p*-values. *, **, and *** indicate statistical significance at p<0.05, p<0.01, and p<0.001, respectively.

**Table 7:** Performance on SEED dataset when reconstruction loss, contrastive loss, MMD alignment, and classifier input are changed jointly.

Loss Functions and Classifier Input	Cross-Subject				Cross-Session			
	Acc ± Std (%)	F1 (%)	Precision (%)	Recall (%)	Acc ± Std (%)	F1 (%)	Precision (%)	Recall (%)
μ	**95.01 ± 06.67**	**94.65**	**96.06**	**94.95**	**96.84 ± 04.49**	**96.74**	**97.46**	**96.80**
$\sigma^{2}$	89.81 *** ± 07.86	89.44	91.48	93.03	94.39 *** ± 06.60	94.12	95.23	94.31
$\mu +\sigma^{2}$	89.43 *** ± 08.50	88.76	90.97	89.34	92.60 *** ± 07.69	92.17	93.89	92.52
$\left[\mu ,\sigma^{2}\right]$	89.36 *** ± 07.46	88.74	91.07	89.25	92.44 *** ± 07.90	92.17	93.32	92.38

The best results are highlighted in bold. The Wilcoxon signed-rank test was used to compute the *p*-values. *, **, and *** indicate statistical significance at p<0.05, p<0.01, and p<0.001, respectively.

## Data Availability

This study uses publicly available, established datasets (SEED, SEED-IV). The SEED dataset is available at https://bcmi.sjtu.edu.cn/home/seed/seed.html (accessed on 8 May 2015), and the SEED-IV dataset is available at https://bcmi.sjtu.edu.cn/home/seed/seed-iv.html (accessed on 8 February 2018).

## Abbreviations

The following abbreviations are used in this manuscript:

EEG	Electroencephalogram;
SCVAE-Net	Supervised Contrastive Variational AutoEncoder Network;
DA	Domain Adaptation;
VAE	Variational Autoencoder;
MMD	Maximum Mean Discrepancy;
CNNs	Convolutional Neural Networks;
RNNs	Recurrent Neural Networks;
GANs	Generative Adversarial Networks;
MK-MMD	Multi-Kernel Maximum Mean Discrepancy;
KL	Kullback–Leibler;
GAP	Global Aaverage Pooling;
CA	Channel Attention;
ReLU	Activation Function;
RKHS	Reproducing Kernel Hilbert Space;
DE	Differential Entropy;
LOSO	Leave One Subject Out.
